# Mathematical Modeling of the Role of Mitochondrial Fusion and Fission in Mitochondrial DNA Maintenance

**DOI:** 10.1371/journal.pone.0076230

**Published:** 2013-10-11

**Authors:** Zhi Yang Tam, Jan Gruber, Barry Halliwell, Rudiyanto Gunawan

**Affiliations:** 1 Institute for Chemical and Bioengineering, ETH Zurich, Zurich, Switzerland; 2 Department of Biochemistry, Centre for Life Sciences, National University of Singapore, Singapore, Singapore; University of Nottingham, United Kingdom

## Abstract

Accumulation of mitochondrial DNA (mtDNA) mutations has been implicated in a wide range of human pathologies, including neurodegenerative diseases, sarcopenia, and the aging process itself. In cells, mtDNA molecules are constantly turned over (i.e. replicated and degraded) and are also exchanged among mitochondria during the fusion and fission of these organelles. While the expansion of a mutant mtDNA population is believed to occur by random segregation of these molecules during turnover, the role of mitochondrial fusion-fission in this context is currently not well understood. In this study, an *in silico* modeling approach is taken to investigate the effects of mitochondrial fusion and fission dynamics on mutant mtDNA accumulation. Here we report model simulations suggesting that when mitochondrial fusion-fission rate is low, the slow mtDNA mixing can lead to an uneven distribution of mutant mtDNA among mitochondria in between two mitochondrial autophagic events leading to more stochasticity in the outcomes from a single random autophagic event. Consequently, slower mitochondrial fusion-fission results in higher variability in the mtDNA mutation burden among cells in a tissue over time, and mtDNA mutations have a higher propensity to clonally expand due to the increased stochasticity. When these mutations affect cellular energetics, nuclear retrograde signalling can upregulate mtDNA replication, which is expected to slow clonal expansion of these mutant mtDNA. However, our simulations suggest that the protective ability of retrograde signalling depends on the efficiency of fusion-fission process. Our results thus shed light on the interplay between mitochondrial fusion-fission and mtDNA turnover and may explain the mechanism underlying the experimentally observed increase in the accumulation of mtDNA mutations when either mitochondrial fusion or fission is inhibited.

## Introduction

Mitochondria are the powerhouses of eukaryotic cells, whose main function is to produce ATP [1]. Mitochondria also possess their own genome, mitochondrial DNA (mtDNA), which encodes several key proteins involved in ATP production. In contrast to nuclear DNA (nDNA), a single eukaryotic cell can harbor 1,000 s of mtDNA [2] and mtDNA are continuously turned over, independent of the cell cycle [3]. The turnover of mtDNA occurs through replication and degradation of these molecules, which happen along with the biogenesis and autophagy of mitochondria organelles. Mutations in mtDNA can occur during replication and thus mtDNA molecules in a single cell may not share the same sequence, a condition called heteroplasmy.

Mitochondrial DNA mutations can lead to the loss of mitochondrial function when the level of mutations exceeds a critical threshold [4], [5]. Such mutations have been implicated in a wide range of human pathologies such as sarcopenia, and even ageing [6]. While the mechanism underlying accumulation of mutant mtDNA is not precisely understood, the consensus is that such mutations expand over time due to random segregation during mtDNA turnover [7]. Consistent with this hypothesis, the proportion of cells with heteroplasmic mtDNA has been reported to significantly increase with age [8]. Interestingly, aged cells have also been found to harbor high fraction of the same mutations (>80%) [9], [10], an observation that is usually explained by clonal expansion of a single original mutation event. Hence, understanding how mtDNA mutations propagate and clonally expand in cells is critical in elucidating the pathogenesis of mitochondrial diseases as well as the ageing process.

The mtDNA random segregation hypothesis has been implemented by us and others in computer simulations, which were able to reproduce not only the increase of heteroplasmy frequency [11], [12], but also the random occurrences of clonal expansion from a single mutational event [13]. In these studies, however, the mtDNA population has typically been assumed to be well-mixed. Mixing of mtDNA is a result of mitochondrial fusion-fission, a process in which mitochondria fuse forming a larger organelle and a mitochondrion divides to form two separate organelles, respectively [14]. Perturbations of mitochondrial fusion and fission have been shown experimentally to affect mitochondrial morphology and functions [15]. Inhibition of either fusion or fission has also been observed to cause a rapid accumulation of deleterious mtDNA mutations and loss of mitochondrial functions in mice and cell culture studies [16], [17]. The random segregation hypothesis and previous computational models cannot immediately explain these observations, giving motivation to investigate the role of mitochondrial fusion-fission on the maintenance of mtDNA.

Modeling of mitochondrial fusion-fission process has recently received more attention in the literature. A model of mitochondrial fusion-fission process has been developed to study the expansion of damaged mitochondrial components, suggesting that there exists an optimal fusion-fission frequency for maintaining mitochondrial function [18]. This model assumed that (1) mitochondria population are well-mixed (i.e. ignoring spatial distribution) and (2) mitochondria exist only in two states due to fusion-fission: fused or solitary. In another study, the accumulation of infectious molecular damage was modeled using a probabilistic approach. As fusion-fission was assumed to propagate the infectious damage, the model expectedly predicted that a deceleration in the fusion-fission cycle could delay the loss of mitochondria quality [19]. In the present study, we have created a stochastic (probabilistic) model of mitochondrial fusion-fission and mtDNA turnover, accounting for spatial and size distribution of mitochondria. The spatial position of mitochondria constrains mitochondrial fusion events such that this process could only occur between neighboring mitochondria. In addition, a wide distribution of mitochondrial sizes has been reported in the literature [20], which carries important information regarding the relative balance between fusion and fission. Such data will be of value in elucidating mechanistic aspects of these processes. The effects of fusion-fission dynamics on the clonal expansion of mtDNA mutations and its role in the maintenance of mitochondrial genome were investigated here by performing model simulations of a cell population at different fusion-fission rates or with an unbalanced fusion or fission process.

## Methods

The present model was developed to simulate the distribution of two mtDNA sequences (genotypes), wild-type (*W*) and mutant (*M*), in mitochondria within a cell and among cells in a population (tissue). In the model, the distribution of the two mtDNA genotypes was governed by random events involved in mtDNA turnover (i.e. replication and degradation) and mtDNA mixing through mitochondrial fusion-fission. In this case, the probability of any random event *j* to occur between time *t* and *t*+*dt* was proportional to the time window *dt* and the propensity function *a_j_*(**X**(*t*)). The state vector **X**(*t*) comprised the number of mitochondria and the distribution of mtDNA molecules in individual mitochondria. Because of random mtDNA turnover and mitochondrial fusion-fission, the fractions of *M_i_* and *W_i_* in the *i-*th mitochondrion (for *i = *1, 2, …, *N_mito_*) and the number of mitochondria *N_mito_* are expected to stochastically deviate over time among cells in the population, even in cells that start with identical initial states. Importantly, not only the overall fraction of *M* in each cell (R_M_^cell^), but also the proportion of *M* in each mitochondrion of a cell (R_M,i_^mito^) will dynamically vary. When R_M_^cell^ is 0 or 1, the cell harbors homoplasmic wild-type or mutant mtDNA, respectively; otherwise, the cell holds a heteroplasmic mixture of wild-type and mutant mtDNA. The cell-to-cell variation in R_M_^cell^ is referred to as intercellular mtDNA heterogeneity, while the variation of R_M_^mito^ among mitochondria in a single cell is called inter-mitochondrial mtDNA heterogeneity. In this case, the increase in inter-cellular mtDNA heterogeneity over time (age) among cells is called mtDNA random genotypic drift.

**Figure 1:**
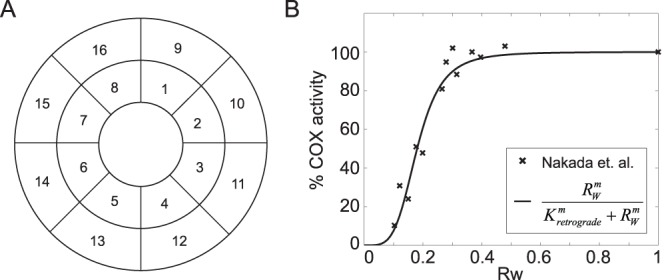
Two dimensional representation of the cell and the retrograde signaling function. (A) A two dimensional representation of the cell partitioned into 16 compartments. (B) The retrograde signaling is modeled according to the COX activity response to a decrease in the wild-type mtDNA fractions in mouse cybrid cells carrying a mixture of wild-type and mutant mtDNA with a pathogenic 4696 bp deletion mutation. The parameters were obtained using least square estimation to the data.

To account for the spatial distribution of mitochondria in a cell, a 2D compartmental cell model was considered with a diameter of 32 µm and a nuclear (inner) diameter of 20 µm, based on a cross sectional image of mammalian cells [21]. Each cellular compartment was sized according to the maximum distance of 3 µm that is traveled by a single mitochondrion in a directed movement event (ignoring 2 data points at ∼6 µm) [22], leading to an approximate partitioning of the 2D cell into 16 compartments (see Fig. 1 *A*). In this model, the directed movement of mitochondria was coupled to mitochondria fusion, such that a displacement occurred only when a mitochondrion undergoes fusion with another in an adjacent compartment. The model was simulated using the stochastic simulation algorithm [23], implemented in C++/MPI and performed on a parallel high performance computing cluster. The Mersenne Twister algorithm was used for the generation of random numbers [24]. Model simulation results presented in the next section were taken from the stochastic simulations of 10,000 cells.

While competing replication models have been proposed to explain how nucleoids mediate mtDNA inheritance [25], [26], recent evidence has pointed to the “faithful nucleoid” model, where there is no mixing of mtDNA among nucleoids [27]. In the model, we track the number and genotype (*W* or *M*) of the nucleoids in individual mitochondria. Also, *de novo* mutation rate was set to zero, as our previous computational study and a recent deep sequencing of mouse mtDNA suggested that such mutations mostly occur during development, with little somatic contribution [11], [28]. In the model simulations, cells were initialized with 80 mitochondria, each containing 4 nucleoids [29], [30]. The mitochondria were randomly placed in the cell compartments. Unless indicated differently, mutant and wild type nucleoids were uniformly randomly distributed across the mitochondrial population. Details on how each mitochondrial process is modeled and how the parameter values are determined, are presented below.

### Nucleoid Replication and Mitochondrial Autophagy

In the simulations of neutral mutations, the propensity for nucleoid replication is equal to *a_R,_*_0_, a constant that was set such that replications will balance degradation at steady state (see Table 1). On the other hand, the degradation of nucleoids was done by removing individual mitochondria, mimicking mitochondrial autophagy (mitophagy), whose propensity is computed as:
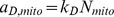
where *N_mito_* is the total number of mitochondria in the cell, a value that will vary with time. Unless indicated otherwise, every mitochondrion has equal probability to be removed by mitophagy. The parameter *k_D_* was determined from the half-life of mitochondrial DNA 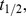 specifically 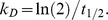 While there exist great discrepancies in the reported half-lives of mitochondria, with values ranging from 2 days to 2 years [31]–[35], an intermediate half-life of 30 days was used in this study. A time-scaling can be done to translate the simulation results to a different half-life value, if desired.

**Table 1:** Model Parameters.

Parameter	Value	Remarks
**Mitochondrial autophagy**		
*k_D_*	0.023 day^−1^	For 30 days half-life [50]
**Replication**		
*a_R,0_*	7.4 day^−1^	This value equals to *k_D_* multiplied by the steady state number of nucleoids ( = 320).
*r_max_*	15	*r_max_* +1 is the maximum retrograde amplification of mtDNA copy number, reported be ∼16 times the basal rate [39].
*K_retrograde_*	0.180±0.007	The level of *R_W_* at the midpoint (50%) amplification, see Fig. 1 *B*
*M*	4.3±0.6	see Fig. 1 *B*
**Mitochondrial fission propensity (** ***τ*** ** = 1 day)**		
*V_F,max_*	8.6 × 10^4^ day^−1^	
*K_F_*	30	
*N*	6.0	
**Mitochondrial fusion propensity (** ***τ*** ** = 1 day)**		
*a_fusion_*	0.123 mitochondrion^−1^ day^−1^	

**Figure 2:**
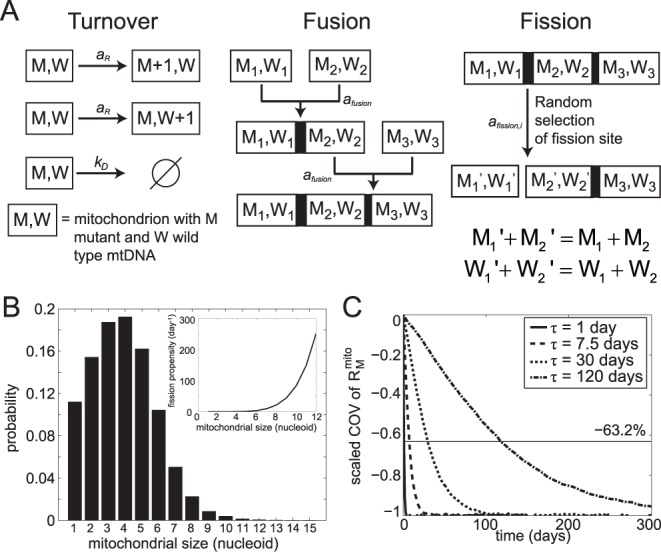
Mitochondrial fusion-fission model. (A) During a mitochondrial fusion, the nucleoid information (*W* and *M*) of the precursor mitochondria is retained and a fission site is created (bold line). During fission of a previously fused mitochondrion, a fission site is randomly chosen from the possible sites in the mitochondrion selected for fission. The redistribution of nucleoid contents between the two daughter mitochondria is determined randomly according to a Binomial distribution, while the particular nucleoids to be transferred are randomly taken from a Hypergeometric distribution. During fission of a primary mitochondrion, i.e. mitochondrion without any fission site, nucleoids are randomly distributed between two daughter mitochondria. (B) Steady state distribution of mitochondrial size as a function of mitochondrial size. In the figure inset, the fission propensity is shown as a function of mitochondrial size (number of nucleoids). (C) Mitochondrial fusion-fission and nucleoids mixing rate. Mitochondrial heterogeneity in each cell is represented by the mean coefficient of variation (COV) of *R_M_^mito^*. The mean COV of R_M_^mito^ is scaled such that the steady state value is −100%. In this case, the mixing time *τ* is defined as the time for the scaled COV of R_M_^mito^ to reach −63.2%. A faster decrease in the mean COV of R_M_^mito^ indicates a faster mixing and hence is indicated by a smaller mixing time constant *τ*. Simulations were performed without mtDNA turnover.

The accumulation of deleterious mutant mtDNA can have a negative impact on cellular respiration. In such a case, the cell can trigger a nuclear response that upregulates mitochondrial biogenesis (mitogenesis) and mtDNA replication, a process known as retrograde signalling [1]. Mitochondrial mass and mtDNA copy number have been shown to increase in tandem with higher mtDNA mutation burden in mitochondrial myopathies [9], [36], [37]. In the model, the retrograde response was simulated by increasing the nucleoid replication propensity in response to mutant mtDNA accumulation, according to:
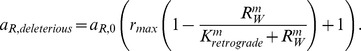



The variable *R_W_* above is the average fraction of wild-type nucleoid among mitochondria (i.e. the average of (1−R_M,i_^mito^)) in a cell. The sigmoidal function is motivated by the activity data of cytochrome c oxidase (COX) as a function of the relative proportion of wild-type and mutant mtDNA in cybrid cells (see Fig. 1*B*) [38]. COX is an enzyme complex involved in the mitochondrial ATP production and its activity is used as an indicator of mitochondrial respiration function. Based on the equation above, the maximum amplification of mtDNA replication by retrograde signaling (at *R_W = _*0) is *r_max_* +1, which has been reported to be ∼16 times the basal rate [39]. A linear function can also be used in place of the sigmoidal function above, without changing the general trend and conclusions from the model simulations (see Results and Discussion section). The implementation of mtDNA turnover process is illustrated in Fig. 2 *A*.

### Mitochondrial Fusion-fission

Fusion was assumed to occur only between mitochondrial pairs that reside in the same or in adjacent cellular compartments. The propensity of fusion between each feasible mitochondrial pair was set to a constant value *a_fusion_*, given in Table 1, which was determined based on the rate of mixing of mitochondrial bound proteins (see further details below). In other words, the fusions of mitochondria were assumed to be size-independent. In a fusion event, two mitochondria were randomly selected from the set of feasible pairs; the nucleoids of one of the mitochondria were then transferred to the other; and the empty mitochondrion was subsequently removed. During the duplication process, a fission site was also created to preserve the original nucleoid distributions from each precursor mitochondrion (see Fig. 2 *A*).

When a fission event occurred to a previously fused mitochondrion, a random fission site was selected and the nucleoid contents of the two mitochondrial subcompartments adjacent to the fission site, arbitrarily called compartment A and B, were exchanged. The nucleoids assigned to compartment A were randomly generated using a Binomial distribution with a mean of *Np*, where *N* is the total nucleoids in compartment A and B and *p* is equal to the ratio between the nucleoids in A and *N*. When this random number differed from the original nucleoid count in A, the exchange of nucleoids was randomly sampled from a Hypergeometric distribution, such that mutant and wild-type nucleoids are equally likely to be exchanged. A majority of fusion-fission events have been shown to occur transiently, a process called kiss-and-run, where a fusion event was followed immediately with a fission near or at the site of fusion [40]. While mitochondrial matrix content could mix during this process, there was little or no mixing of mitochondrial membrane-bound proteins and nucleoids. By sampling from Binomial distribution as done above, the number of nucleoids exchanged between compartments will be small, such that most fissions would lead to little or no exchange of nucleoids, mimicking kiss-and-run fusion-fission. However, once in a while, a large number of nucleoids were transferred, which simulated non-transient fusion-fission events. In a previous modeling of mitochondrial fusion-fission [18], a small fixed number of mitochondrial functional units (2 out of 10) were exchanged during fusion-fission events. However, we have further used two sources of information to calibrate the frequency of fusion-fission events: the size distribution of mitochondria and the mixing times of mitochondrial membrane-bound proteins, as discussed below. Finally, one of the daughter mitochondria was placed in the original cellular compartment, while the other was randomly placed in either the same or in neighboring cellular compartments.

In the following, mitochondria that do not possess any fission site are referred to as primary mitochondria. In this case, fissions were assumed to occur along the length of the mitochondria with equal probability. The size of one of the daughter mitochondria was determined by taking a random integer from a discrete uniform distribution, ranging between 1 and the size of the mitochondrion selected for fission. The other daughter mitochondrion was assigned the remaining nucleoids, i.e. those not assigned to the daughter mitochondrion above. The number of mutant (or wild-type) nucleoids in the daughter mitochondria was again determined by a random sample from the Hypergeometric distribution. Finally, the random sampling of the nucleoid content of daughter mitochondria was constrained such that no empty mitochondrion is generated, which was done by setting the smallest possible mitochondria size to 1 nucleoid. This constraint is consistent with an observation that mitochondria contain at least one nucleoid during transition to a fragmented morphology [41]. Figure 2 *A* illustrates the implementation of the fusion-fission process in this model.

Mitochondria have been reported to contain roughly between 1 and 10 nucleoids and between 1 and 15 mtDNA molecules [42]. The reported distributions of mitochondrial sizes in the literature differ, from geometric [30] to unimodal [21] and multimodal distribution [20], but agree that larger mitochondria are less frequent than smaller ones, possibly due to increased likelihood to undergo fission. Correspondingly, the probability of a mitochondrion undergoing fission has been proposed to increase with its length [43]. In the model, the fission propensity of the *i-*th mitochondrion was assumed to increase with its size (i.e. nucleoid content), such that:
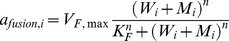
where 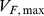 denotes the maximum fission propensity. The sigmoidal dependence of fission on the nucleoid content of mitochondria was selected in order to reproduce a unimodal mitochondrial nucleoid counts. The use of a linear fission propensity gave a geometric size distribution where most mitochondria possess 1–2 nucleoids (see Fig. S1), but still provided the same general observations on the effect of fusion-fission on mtDNA random drift (see further discussion in the next section). The values of 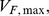 *K_F_* and *n* are given in Table 1, which were determined according to the procedure described below.

The fission parameters were adjusted such that each mitochondrion contains between 1 to 10 nucleoids per mitochondrion and the majority of mitochondria possess 3–4 nucleoids. In this case, mitochondrial fusion-fission simulations were performed without mtDNA turnover and using cells initiated with 80 mitochondria and 4 wild-type nucleoids, as before. The total number of nucleoids will therefore remain the same, removing the effect of nucleoid count on the mitochondrial size distribution. Since the steady state mitochondrial size distribution depends on the balance between the fusion and fission process, 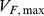 was set as an unknown multiple of *a_fusion_*, whose value was manually changed along with *K_F_* and *n.* By doing so, *a_fusion_* will determine how fast the mitochondrial population achieves steady state, but will not affect the size distribution. Fig. 2 *B* shows the simulated size distribution of mitochondria at steady state for the values reported in Table 1, where most mitochondria contain 3 to 5 nucleoids and large mitochondria with 11 nucleoids or more are rarely encountered.

By keeping 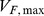 as a constant multiple of *a_fusion_*, the fusion parameter *a_fusion_* influences the overall fusion-fission frequency and thus the mixing rate of nucleoids among mitochondria. While the frequency of fusion-fission (involving complete fusions) is currently not known, data on the mixing of mitochondrial components from fused cells are available. For example, differentially labeled mitochondrial membrane-bound protein in fused HeLa cells took more than 1 day to become well-mixed (see Fig. 3 in [44]). Also, normal mitochondrial morphology and function in fusion of two mouse cells, each containing distinct pathogenic mtDNA mutations, was restored after 10–14 days [45]. To determine the parameter *a_fusion_*, cells were again initiated with 80 mitochondria each containing 4 nucleoids. Subsequently, nucleoids of mitochondria located in one half of the cells were labeled as W and the other half as M, giving an R_M_^cell^ of 0.5 and R_M_^mito^ of either 0 or 1. In order to relate *a_fusion_* with the rate of mixing of mtDNA, a metric called *mixing time constant* or *τ* is used. The mixing time constant *τ* is defined as the time for the coefficient of variation (COV) of R_M_^mito^, averaged over the cell population, to reach 63.2% of its final steady state change. This time constant *τ* is analogous to the characteristic rise time of a first-order linear time invariant (LTI) model 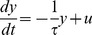 with a zero initial condition (*y*(*t*_0_) = 0), in response to a unit step function of *u* (i.e. *u*(*t*) = 1 for *t* ≥0, otherwise *u*(*t*) = 0). By adopting this definition, the time to reach a prescribed percentage of the steady state change can be pre-computed, for example such a time for 98% is 4*τ*. Figure 2 *C* shows the results of the stochastic simulations for different *a_fusion_* values, where the time-profiles of the mean COV of R_M_^mito^ resembled a typical unit step response of the LTI system mentioned above. In this case, a lower mixing time constant refers to a faster mixing rate due to more frequent fusion-fission events (higher *a_fusion_*). The value of *a_fusion_* corresponding to a mixing time constant of 1 day is given in Table 1.

**Figure 3:**
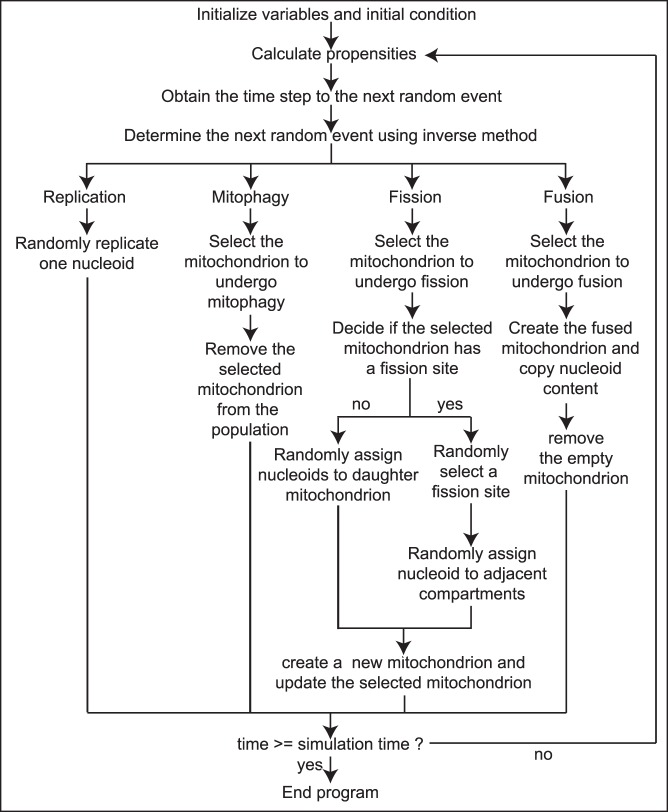
Pseudo-code of the stochastic simulation algorithm implementation of the present model.

The pseudo-code of the model implementation is shown in Fig. 3 and the source code of the model is available in the Code S1. After initialization, the total propensity of all events is computed, i.e. the sum of the propensities of mtDNA replication (*a_R,_*_0_ or *a_R,deleterious_*), mtDNA degradation (*a_D,mito_*), mitochondrial fusion (the sum of *a_fusion_* over all feasible mitochondria pairs) and fission (the sum of *a_fission,i_* over all mitochondria). The time to the next reaction is then randomly generated based on the total propensity, which is then continued with the random assignment of the particular event, following the original SSA procedure [23]. Validation simulations of our model implementation produced the expected general behavior (see Fig. S2). Briefly, in the case of neutral mutations, the total mutation burden stochastically varied with time, but the average remained relatively constant. Nevertheless, cells can harbor a large fraction of mutations (clonal expansion) as a result from such stochastic behavior without any increase in the total mutation burden (see Fig. S2 *B*). For deleterious mutations, retrograde signalling has the effect of preventing the clonal expansion of mutations by effectively increasing mitochondrial population and thereby reducing stochasticity of the mitochondrial genotypic random drift (see Fig. S3).

## Results and Discussion

**Figure 4:**
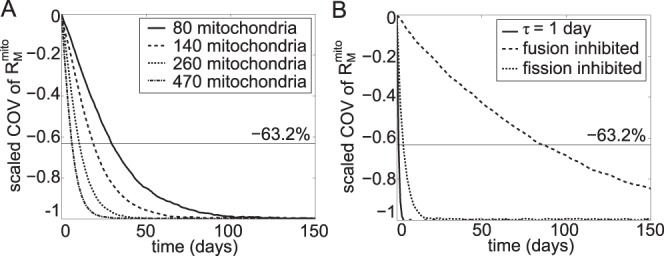
Simulations of mitochondrial fusion-fission without mtDNA turnover. The mean COV of R_M_^mito^ depends on the number and size distribution of mitochondria, and is therefore scaled such that the steady state value is −100%. In this case, the mixing time *τ* is defined as the time for the scaled COV of R_M_^mito^ to reach −63.2%. (A) Lower mixing time *τ* is observed when the number of mitochondria becomes larger. (B) Inhibiting mitochondrial fusion or fission alone slows mixing. Simulations were performed by setting the parameter *a_fusion_* or 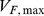 500 times lower than that the values reported in Table 1.

**Figure 5:**
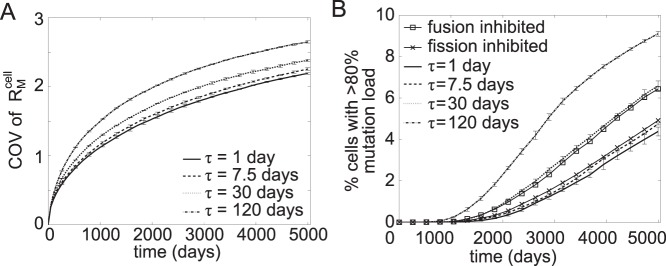
Simulations of mitochondrial fusion-fission with neutral mutations. The simulations were done in triplicate and the error bars show the standard deviation. (A) The COV of R_M_^cell^ increases at a slower rate with decreasing mixing time constant (i.e. faster fusion-fission). (B) The inhibition of fusion or fission and slower fusion-fission lead to an increase in the rate at which cells reach 80% mutation level. Simulations were performed with an initial R_M_^cell^ of 10% mutation load in the presence of mtDNA turnover.

By varying the overall fusion-fission frequency (i.e. changing *a_fusion_* and 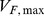 by the same relative amount), simulations of neutral mutations without mtDNA turnover showed that, as expected, faster fusion-fission results in a better mixing of nucleoids in cells (see Fig. 2 *C*), as indicated by the lower mixing time constants. The frequencies of fusion and fission events per mitochondrion per day for different mixing time constants are given in Table 2. Similarly, when the number of mitochondria in the cell increases, the total propensity for fusion and fission events will rise and this correspondingly leads to a faster mixing of nucleoids (see Fig. 4 *A*). In the presence of mtDNA turnover, slower mtDNA mixing coincided with higher mutation burden variability among cells (see Fig. 5 *A*) and more cells accumulated mutations higher than 80% within a given time period (see Fig. 5 *B*). In other words, slower fusion-fission has the effect of quickening the random segregation of cells into homoplasmy W or M, as well as the process of clonal expansion. To explain this phenomenon, consider a hypothetical scenario where two mitophagic events occur sequentially in two cells with different inter-mitochondrial mtDNA heterogeneity, without fusion-fission mixing. In the cell with uniform R_M_^mito^, random mitophagy will not lead to different R_M_^cell^ fates over time, while in the cell with more heterogeneous R_M_^mito^, the same random mitophagy events can produce very different R_M_^cell^ outcomes (see Fig. S4). Hence, imperfect mixing of mtDNA nucleoids can give rise to additional variability in the outcome of mitophagy events, and in this case, higher inter-mitochondrial mtDNA heterogeneity of mitochondria population contributes toward an increase in the intercellular mtDNA heterogeneity (see Fig. 5 *A*).

**Table 2:** Fusion-Fission Frequency at Different Mixing Time Constants.

Conditions	Frequency of fusion-fission events (per mitochondrion per day)
***τ*** ** = 1 day**	7.6
***τ*** ** = 7.5 days**	1.0
***τ*** ** = 30 days**	0.26
***τ*** ** = 120 days**	0.06
**Fusion Knock-down**	0.04
**Fission Knock-down**	3.0

**Figure 6:**
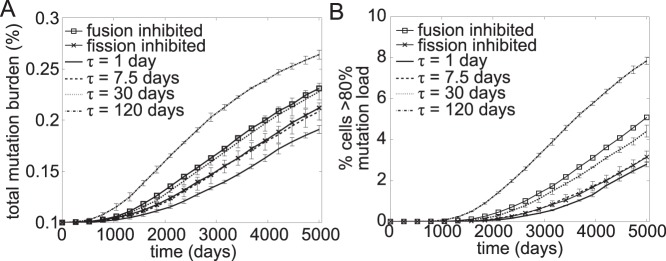
Simulations of mitochondrial fusion-fission with deleterious mutations. The simulations were done in triplicate and the error bars show the standard deviation. (A) Inhibiting fusion or fission separately and slowing down fusion-fission quicken the accumulation of total mutation burden. (B) Similarly, the rate at which cells reach 80% mutation level increases with slower fusion-fission mixing of nucleoids. However, for the same mixing time constant, retrograde signalling reduces the rate at which cell undergo clonal expansion. Simulations were performed with an initial R_M_^cell^ of 10%.

In the case of deleterious mutations, model simulations showed that the total mutation burden in the cell population increased with time (see Fig. 6 *A*), since clonal expansion of these mutations triggers retrograde signalling, increasing mtDNA copy number in cells that predominantly carry mutant population. Similar to the case of neutral mutations, decreasing fusion-fission rates caused more cells to reach high level of R_M_^cell^ (see Fig. 6 *B*) and consequently, the total mutation burden increased more rapidly with slower mtDNA mixing. The same explanation discussed above applies, where the increase of inter-mitochondrial mtDNA heterogeneity due to slower fusion-fission promotes clonal expansion. On the other hand, comparing the frequencies of clonal expansion of neutral and deleterious mutations in Figs. 5 *B* and 6 *B* shows that retrograde signalling can lower the fraction of cells with clonally expanded deleterious mutations. While this beneficial effect of retrograde response has been previously reported [12], our simulations further suggest that this effect lessens with the rate of fusion-fission.

**Figure 7:**
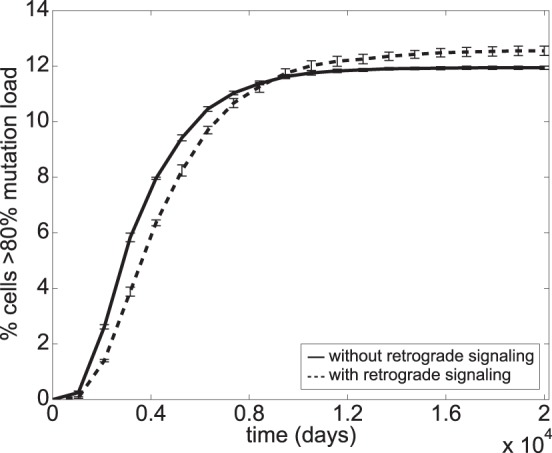
Amplification of clonal expansion by retrograde signalling when mitochondrial fusion-fission is slow. The simulations were done in triplicate using *τ = *120 days and an initial R_M_^cell^ of 10%. The error bars show the standard deviation.

In general, the reduction of random clonal expansion by the actions of retrograde signalling is an indirect result of an increase in mtDNA population, which not only lowers the stochasticity that drives the mtDNA random genotypic drift, but also leads to more efficient mixing of mtDNA as illustrated in Fig. 4 *A*. However, retrograde signalling also indirectly triggers faster mtDNA turnover and thus more frequent mitophagy. To the best of our knowledge, there is no evidence indicating that retrograde signalling directly regulates the activity of mitochondrial autophagy or fusion-fission. In this case, as long as fusion-fission can keep nucleoid populations relatively well-mixed in between mitophagy events, the benefit of retrograde signalling in lowering clonal expansion can be realized. But, when fusion-fission is slow, a faster mitophagy means higher inter-mitochondrial mtDNA heterogeneity and increased stochasticity, as there is less time for fusion-fission mixing in between mitophagy events. Indeed, extended model simulations of cells harboring deleterious mutations demonstrated that the retrograde response could not reduce and may even slightly increase the frequency of clonal expansion when fusion-fission was inefficient (*τ = *120 days), as shown in Fig. 7. Thus, the protective effect of the nuclear retrograde signalling against clonal expansion relies critically on an unimpaired fusion-fission process.

In the model simulations, lowering either fusion or fission individually (i.e. changing *a_fusion_* and 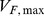 independently) by 500 times from those for *τ* = 1 day, led to slower fusion-fission process and mixing of nucleoids, as shown in Table 2 and Fig. 4 *B*. This is because, when the fusion rate is lowered, cells contain fewer large mitochondria and consequently experience fewer fission events. On the other hand, decreasing mitochondrial fission frequency causes a drop in the mitochondrial count in the cell, which in turn leads to fewer fusion pairs and less frequent fusion events (see Table 1). Following the explanation above, the lesser mixing associated with a knock down of fusion or fission also means faster random segregation of cells and clonal expansion of mtDNA mutations, regardless of the type (silent or deleterious). Again, retrograde response became less efficient in slowing down clonal expansion when the capacity of fusion or fission was negatively affected (see Figs. 5 *B* and 6 *B*).

Mitochondria with a lower membrane potential have been shown to be prevented from fusion with others and preferentially autophagosized (selective degradation) [46], acting as a mechanism to selectively remove deleterious mtDNA mutations. When the degradation propensity for dysfunctional mitochondria with an R_m_^mito^ >0.8 was set 20% higher than the rest (i.e. increasing the propensity of degradation of these mitochondria by 20%), simulations showed that no clonal expansion occurs and the cell population eventually becomes homoplasmic wild-type (see Fig. S5). Nevertheless, clonal expansion of deleterious mtDNA mutations is frequently observed experimentally with age and in age-related ailments, such as sarcopenia and neurodegenerative diseases, suggesting that such a surveillance mechanism, like the fusion-fission process, weakens with age. When both selective degradation and fusion-fission become ineffective, clonal expansion of deleterious mutations can then proceed, even in the presence of retrograde signalling as explained above.

The simulation results above could be scaled to different mtDNA turnover rates and mixing time constants by scaling the time axis. For example, if the mtDNA turnover rate is adjusted to a half-life of 2 days (from 30 days), the results of such simulations will be equivalent to those of this study with a different time axis, one that is scaled by 1/15. In addition, the trend of faster random segregation of cells with increased fusion-fission remained valid for a linear size-dependent fission function that generates a geometric mitochondrial nucleoid distribution (see Fig. S1) and did not change with the use of a different cellular shape and compartmentalization (a cylindrical cell, see Fig. S6). Similarly, the same observations about the actions of retrograde signalling could be reproduced using a linear retrograde function (see Fig. S7), where the replication propensity increases linearly when the number of wild-type nucleoids falls below a given set point [12]. Finally, the model simulations were done using a steady state nucleoid population of 320 (or 80 mitochondria). An increase in the nucleoid population would increase both the frequency of mitochondrial turnover and fusion-fission, leading to a better mixing of nucleoids (see Fig. S2 C). Analogous to the action of retrograde signalling, cells with more nucleoids and mitochondria are expected to be less susceptible to mutant mtDNA clonal expansion, provided that the fusion-fission can provide an effective mixing of nucleoids.

Here, we have ignored the occurrence of *de novo* mutations. In the presence of *de novo* mutations, the mean of R_M_^cell^ is expected to increase with time (age). The rate of increase in the mean of R_M_^cell^ will be proportional to the mtDNA turnover and the mutation rate [11] using the assumption that *de novo* mutation arises from mtDNA replication error. Nevertheless, when fusion and/or fission rate is lowered, the corresponding increase in stochasticity will enhance the frequency of clonal expansion in this case. In addition, we also expect our basic conclusion to stay valid in a 3D cell. If the parameter values are kept the same as in the simulations above, a 3D cell lattice will lead to larger pairwise combinations of mitochondria and thus to more frequent fusion-fission events and faster mixing of nucleoids. The differences between 2D and 3D lattice simulations will be less pronounced when the parameters for the fusion-fission propensities are recalibrated for the 3D cell (by the same manner as described in Method section).

Mitochondrial DNA mutations are often associated with diseases involving cell types with unique morphological and structural characteristics. For example, neurons have long polarized cell bodies, and skeletal and cardiac muscle tissues have internal structures (fiber striations) that limit mitochondrial movements. While our observations above were based on simulations using spherical and cylindrical cell morphology, the conclusion that higher heterogeneity of nucleoid (mtDNA) population can induce faster segregation of mtDNA genotype among cells, should also be applicable in other cellular contexts. In general, any restriction on mitochondria movement and localization would slow the mixing of nucleoids. Based on our simulations, such circumstance could lead to more frequent clonal expansion of mutant mtDNA molecules. While the model was not originally created to make quantitative prediction of mtDNA mutations in ageing and in diseases, the insights from model simulations are consistent with experimental observations from diseases related to mtDNA mutations. A more accurate model for a specific condition or disease can be created based on the model presented here, if quantitative data on mitochondrial turnover and fusion-fission process are available for the kinetic parameter estimation.

Also, we have assumed that mitochondria movement is associated with mitochondrial fusion and fission, as these events would lead to the mixing of nucleoids. A previous study has shown that mitochondrial movement consists of a Brownian diffusion, interspersed with frequent and brief directed motion [22]. In the model, mitochondria displacements occurred during fusions involving mitochondria from adjoining compartments or during fissions that lead to a placement of a daughter mitochondrion in an adjacent compartment. Consequently, the fusion-fission induced mixing of mtDNA in the model simulations above may underestimate reality. The model can certainly be extended to take into account long-range motility of mitochondria by simulating movements of mitochondria that are independent of fusion or by allowing fusions among all possible pairs of mitochondria in the cell and using distance-dependent propensity.

The model also did not account for possible interactions between mtDNA turnover and mitochondrial fusion-fission,that are independent of mtDNA mutation burden and retrograde signalling. For example, a severe imbalance of fusion-fission has been linked with a drop in cellular ATP and mtDNA counts [16], [47], [48], possibly through a reactive oxygen species (ROS) dependent pathway. Also, PINK1/parkin, a known of player in mitophagy, have been shown to regulate mitofusions, which are key proteins in mitochondrial fusion [49]. In mouse skeletal muscle, knock-out of fusion genes led to increased mtDNA deletion mutations and mitochondrial mass, but a lower total mtDNA copy number [16]. However, milder perturbations (knock-down) of fusion or fission did not appear to have a strong effect on mtDNA replication [16], [17]. Therefore, the model assumption above would fail under severe perturbations of mitochondrial processes (e.g., complete absence of fusion or fission). Model simulations of such scenarios are not expected to replicate the reality and outside the scope of this modeling study. The findings above should be used in the context of age-related changes, involving mild to moderate perturbations (e.g. slowing down fusion-fission and knock-down of fusion or fission).

In mice and in human cell culture, moderate inhibition of either mitochondrial fission or fusion has been shown to increase the burden of deleterious mtDNA mutations [16], [17]. In particular, Chen et al. studied mice lacking mitofusin (Mfn)-1 or 2 or both in skeletal muscle tissue (16). While both single mutant mice appeared normal, Mfn-1 deletion led to more prominent changes in mitochondrial morphology than Mfn-2 deletion at 7 weeks of age (Fig. 1G and H (16)). We further note that at this age, the skeletal muscle of both single mutant mice have roughly equal mtDNA copy number and mtDNA mutation burden as the control mice. Thus, the differential change in the mitochondrial morphology was likely an indication that Mfn-1 deletion causes inflicts the fusion process more severely than Mfn-2 deletion in this tissue. In parallel to our simulations, at older age (8–13 months), Mfn-1 mutant mice harbored significantly higher mutation than control and Mfn-2 mutant mice (Fig. 4C (16)). In a different study, a reduction in important fission proteins Drp1 and hFis by RNAi similarly led to higher burden of functional A3243G mtDNA mutation (causing mitochondrial encephalomyopathy, lactic acidosis, and strokelike (MELAS) episodes) in muscle-derived rhabdomyosarcoma human cell line (17). These observations are consistent with our simulations, suggesting that the increase in mitochondrial population due to retrograde signalling can delay the expansion of functional mutations, but this ability depends on mitochondrial fusion-fission capacity.

## Conclusion

In summary, our model simulations suggest an intuitive and novel mechanism that explains the increase in mutant mtDNA accumulation when mitochondrial fusion-fission is perturbed. In general, inefficient fusion-fission leads to increased heterogeneity of mitochondrial genotype in a cell and correspondingly to higher heterogeneity among cells in a population due to the actions of mitophagy. As a consequence of the increase in stochasticity, random clonal expansion of mutations also becomes more frequent with slower fusion-fission. When mutations are deleterious, cells can trigger the retrograde signalling that increases mitochondrial biogenesis and mtDNA population in the cell. While such retrograde response has been reported to reduce clonal expansion, the fusion-fission simulations here suggest that this beneficial effect requires an efficient fusion-fission process. As the mitochondrial fusion-fission capacity likely diminishes with age, so will the protective ability of retrograde signalling against clonal expansion of deleterious mtDNA mutations.

## Supporting Information

Figure S1 **Stochastic simulations of neutral mutations using a linear fission propensity function.**
(DOCX)

Figure S2 **Stochastic random walk of mitochondrial genotypes.**
(DOCX)

Figure S3 **Effects of retrograde signaling on the accumulation of mutations.**
(DOCX)

Figure S4 **Interplay between mitochondrial turnover and fusion-fission.**
(DOCX)

Figure S5 **Selective degradation of damaged mitochondria prevents clonal expansion.**
(DOCX)

Figure S6 **Stochastic simulations of neutral mutations for a cylindrical cell shape, maintaining the same cellular compartment size.**
(DOCX)

Figure S7 **Stochastic simulations of deleterious mutations with a linear retrograde response function.**
(DOCX)

Code S1 (ZIP)
