# Supplementary material for: Mathematical Modeling of the Role of Mitochondrial Fusion and Fission in Mitochondrial DNA Maintenance
Source: PLoS One. 2013 Oct 11;8(10):e76230. doi: 10.1371/journal.pone.0076230 (PMC3795767; doi:10.1371/journal.pone.0076230)
Supplement: Figure S1 — Stochastic simulations of neutral mutations using a linear fission propensity function. (DOCX) [file pone.0076230.s001.docx]

Figure S1 Stochastic simulations of neutral mutations using a linear fission propensity function. (A) Linear fission propensity function. (B) Steady state nucleoid distribution of mitochondria. (C) Random clonal expansion increases with higher mixing time constants (slower fusion-fission). Simulations of 2,500 cells were performed in quadruplet with an initial R_M_^cell^ of 10%. The error bars show the standard deviation.
