# Supplementary material for: Mathematical Modeling of the Role of Mitochondrial Fusion and Fission in Mitochondrial DNA Maintenance
Source: PLoS One. 2013 Oct 11;8(10):e76230. doi: 10.1371/journal.pone.0076230 (PMC3795767; doi:10.1371/journal.pone.0076230)
Supplement: Figure S2 — Stochastic random walk of mitochondrial genotypes. (DOCX) [file pone.0076230.s002.docx]

Figure S2 Stochastic random walk of mitochondrial genotypes.

(A) Among the 10,000 cells that start with the same level of neutral mutations (initial R_M_^cell^ = 1%), R_M_^cell^ dynamics appeared to be unbiased, consistent with the assumptions in the model that neither turnover nor fusion-fission events enforced any bias toward an accumulation of *M* or *W*. The colored lines indicate the number of cells with a particular mutation load, as indicated by the color legend. Cell simulations were performed using an identical initial R_M_^cell^ of 1%. (B) Total mutation load and mean R_M_^cell^ remain approximately unchanged for neutral mutations. The total mutation load was computed as the ratio between the total mutant nucleoids and the total nucleoids in the whole cell population, while the mean R_M_^cell^ was calculated as the population average of R_M_^cell^. The slight difference between the two variables is due to the unequal number of nucleoids in different cells. (C) Normalized coefficient of variations of R_M_^cell^ on day 5000 for: (1) 320 nucleoids compartmentalized into 80 mitochondria, (2) 320 nucleoids in a cell under well-mixed assumption (i.e. no mitochondrial partitioning of nucleoids), and (3) 600 nucleoids compartmentalized into 140 mitochondria. The normalization was done such that the COV of R_M_^cell^ from the case of 320 nucleoids and 80 mitochondria is equal to 1. The coefficient of variation of R_M_^cell^ scaled roughly with $1/\sqrt{n}$where *n* is the nucleoid population size [[1](#_ENREF_1)]. For example, when the number of nucleoids is increased from 320 to 600, the COV of R_M_^cell^ decreased by a factor of$\sqrt{320}/\sqrt{600}=0.73,$ approximately the value that was produced by the model simulations. (D) Because of the random drift, R_M_^cell^ has a non-zero probability of reaching any values between 0 and 1. In this case, once a cell reached mtDNA homoplasmic condition (i.e. all *W* or *M*), it cannot escape from such state. Hence, cells would asymptotically segregate into the two fixed points: homoplasmic *W* or *M*. As expected, the probability to reach either fully *W* or *M* nucleoids at steady state was determined only by the initial fraction of *M* (or *W*) (simulations are done for 1%, 26% and 50% initial mutation load).

**Reference**

1. Capps GJ, Samuels DC, Chinnery PF (2003) A model of the nuclear control of mitochondrial DNA replication. Journal of theoretical biology 221: 565-583.
