# Supplementary material for: Mathematical Modeling of the Role of Mitochondrial Fusion and Fission in Mitochondrial DNA Maintenance
Source: PLoS One. 2013 Oct 11;8(10):e76230. doi: 10.1371/journal.pone.0076230 (PMC3795767; doi:10.1371/journal.pone.0076230)
Supplement: Figure S3 — Effects of retrograde signaling on the accumulation of mutations. (DOCX) [file pone.0076230.s003.docx]

Figure S3 Effects of retrograde signaling on the accumulation of mutations.

(A) In simulations using retrograde signaling (i.e. mutations are deleterious), the mean R_M_^cell^ remained approximately constant over time, similar to the random drift in Figure S2, but the total mutation burden of the population increased with time. Simulations of 10,000 cells were performed in triplicate with an initial R_M_^cell^ of 10%. The error bars show the standard deviation. This observation is in agreement with experimental observations from mitochondrial myopathies [[1](#_ENREF_1),[2](#_ENREF_2),[3](#_ENREF_3)] and also with a previous simulation study using a well-mixed mtDNA assumption [[4](#_ENREF_4)]. The increase in the total mutation burden occurred because mitochondrial replication under nuclear retrograde regulation positively responds to a higher level of deleterious mutations and thus cells with high R_M_^cell^ necessarily contained more mtDNA than those with low level of mutations. (B) The increase in the mitochondrial population in a cell resulted in reduced stochasticity of the R_M_^cell^ dynamics. As a consequence of this reduction, the number of cells that accumulate high level of deleterious mutations (R_M_^cell^ > 80%) was lower than that in simulations of neutral mutations, in agreement with similar simulations using well-mixed mtDNA assumption [[4](#_ENREF_4),[5](#_ENREF_5)]. In addition, cells rarely became homoplasmic mutant. Therefore, by increasing mitochondrial biogenesis, retrograde signaling not only compensates for the reduction in OXPHOS capacity, at least partially [[6](#_ENREF_6)], but also elicits a barrier for cells to reach detrimental level of deleterious mutations. Simulations were performed with fusion-fission parameters corresponding to *τ* of 1 day and an initial mutation load R_M_^cell^ of 1%.

**References**

1. Herbst A, Pak JW, McKenzie D, Bua E, Bassiouni M, et al. (2007) Accumulation of mitochondrial DNA deletion mutations in aged muscle fibers: Evidence for a causal role in muscle fiber loss. Journals of gerontology Series A, Biological sciences and medical sciences 62: 235-245.

2. Wanagat J, Cao Z, Pathare P, Aiken JM (2001) Mitochondrial DNA deletion mutations colocalize with segmental electron transport system abnormalities, muscle fiber atrophy, fiber splitting, and oxidative damage in sarcopenia. FASEB Journal 15: 322.

3. Johnston W, Karpati G, Carpenter S, Arnold D, Shoubridge EA (1995) Late-onset mitochondrial myopathy. Ann Neurol 37: 16-23.

4. Chinnery PF, Samuels DC (1999) Relaxed replication of mtDNA: A model with implications for the expression of disease. American journal of human genetics 64: 1158-1165.

5. Capps GJ, Samuels DC, Chinnery PF (2003) A model of the nuclear control of mitochondrial DNA replication. Journal of theoretical biology 221: 565-583.

6. Rohas LM, St-Pierre J, Uldry M, Jager S, Handschin C, et al. (2007) A fundamental system of cellular energy homeostasis regulated by PGC-1alpha. Proc Natl Acad Sci U S A 104: 7933-7938.
