# Supplementary material for: Mathematical Modeling of the Role of Mitochondrial Fusion and Fission in Mitochondrial DNA Maintenance
Source: PLoS One. 2013 Oct 11;8(10):e76230. doi: 10.1371/journal.pone.0076230 (PMC3795767; doi:10.1371/journal.pone.0076230)
Supplement: Figure S4 — Interplay between mitochondrial turnover and fusion-fission. (DOCX) [file pone.0076230.s004.docx]

Figure S4 Interplay between mitochondrial turnover and fusion-fission. The outcomes of consecutive mitophagies in cells with higher R_M_^mito^ heterogeneity exhibit a higher degree of stochasticity.
