# Supplementary material for: Mathematical Modeling of the Role of Mitochondrial Fusion and Fission in Mitochondrial DNA Maintenance
Source: PLoS One. 2013 Oct 11;8(10):e76230. doi: 10.1371/journal.pone.0076230 (PMC3795767; doi:10.1371/journal.pone.0076230)
Supplement: Figure S5 — Selective degradation of damaged mitochondria prevents clonal expansion. (DOCX) [file pone.0076230.s005.docx]

Figure S5 Selective degradation of damaged mitochondria prevents clonal expansion. In these simulations, mitochondria harboring R_M_^mito^ > 80% are preferentially removed at 20% higher degradation constant *k_D_* than that for the other mitochondria. Simulations of 10,000 cells were performed in triplicate with an initial R_M_^cell^ of 10%. The error bars show the standard deviation.
