# Supplementary material for: Mathematical Modeling of the Role of Mitochondrial Fusion and Fission in Mitochondrial DNA Maintenance
Source: PLoS One. 2013 Oct 11;8(10):e76230. doi: 10.1371/journal.pone.0076230 (PMC3795767; doi:10.1371/journal.pone.0076230)
Supplement: Figure S6 — Stochastic simulations of neutral mutations for a cylindrical cell shape, maintaining the same cellular compartment size. (DOCX) [file pone.0076230.s006.docx]

Figure S6 Stochastic simulations of neutral mutations for a cylindrical cell shape, maintaining the same cellular compartment size. (A) Compartmentalization of the 2D cylindrical cell. (B) Random clonal expansion increases with longer mixing time constants (slower fusion-fission). Simulations of 2,500 cells were performed in quadruplet with an initial R_M_^cell^ of 10%. The error bars show the standard deviation.
