# Supplementary material for: Mathematical Modeling of the Role of Mitochondrial Fusion and Fission in Mitochondrial DNA Maintenance
Source: PLoS One. 2013 Oct 11;8(10):e76230. doi: 10.1371/journal.pone.0076230 (PMC3795767; doi:10.1371/journal.pone.0076230)
Supplement: Figure S7 — Stochastic simulations of deleterious mutations with a linear retrograde response function. (DOCX) [file pone.0076230.s007.docx]

Figure S7 Stochastic simulations of deleterious mutations with a linear retrograde response function ([5](#_ENREF_5)). (A) The linear retrograde signaling function (inset) and replication propensity as a function of wild-type nucleoids using α=(*r_max_*+1)=16, N_opt_=320 and C_0_=*a_R,0_*. (B) Random clonal expansion increases with longer mixing time constants (slower fusion-fission). Simulations of 2,500 cells were performed in quadruplet with an initial R_M_^cell^ of 10%. The error bars show the standard deviation.
